# Delineating Ecological Boundaries of Hanuman Langur Species Complex in Peninsular India Using MaxEnt Modeling Approach

**DOI:** 10.1371/journal.pone.0087804

**Published:** 2014-02-03

**Authors:** Nag Chetan, Karanth K. Praveen, Gururaja Kotambylu Vasudeva

**Affiliations:** 1 Department of Biology, Undergraduate Program, Indian Institute of Science, Bangalore, Karnataka, India; 2 Center for ecological sciences, Indian Institute of Science, Bangalore, Karnataka, India; 3 Centre for infrastructure, Sustainable Transportation and Urban Planning (CiSTUP), Indian Institute of Science, Bangalore, Karnataka, India; University of Florence, Italy

## Abstract

Hanuman langur is one of the widely distributed and extensively studied non-human diurnal primates in India. Until recently it was believed to be a single species - *Semnopithecus entellus*. Recent molecular and morphological studies suggest that the Hanuman langurs consists of at least three species *S. entellus*, *S. hypoleucos* and *S. priam*. Furthermore, morphological studies suggested that both *S. hypoleucos* and *S. priam* have at least three subspecies in each. We explored the use of ecological niche modeling (ENM) to confirm the validity of these seven taxa and an additional taxon *S. johnii* belonging to the same genus. MaxEnt modeling tool was used with 19 bioclimatic, 12 vegetation and 6 hydrological environmental layers. We reduced total environmental variables to 14 layers after testing for collinearity and an independent test for model prediction was done using ENMTools. A total of 196 non-overlapping data points from primary and secondary sources were used as inputs for ENM. Results showed eight distinct ecological boundaries, corroborating the eight taxa mentioned above thereby confirming validity of these eight taxa. The study, for the first time provided ecological variables that determined the ecological requirements and distribution of members of the Hanuman langur species complex in the Indian peninsula.

## Introduction

Species is one of the fundamental units of biodiversity and of great interest to taxonomists, evolutionary biologists, ecologists, and conservationists [1], [2]. In spite of the importance, till recently, there has been little consensus regarding its definition that resulted in numerous species concepts [3]. However, de Queiroz [4] and Pigliucci [5] considered “species” as metapopulation lineages, a concept that attempts to combine various species concepts. Thus, it is generally accepted that species comprise of lineages, although, there still exists inconsistencies regarding how lineages are diagnosed as species [6], [7]. An integrative approach which uses multiple lines of evidence is usually recommended for recognizing evolutionary lineages [8]. Thus, one of the challenges now is to obtain ample evidence to establish a clear demarcation of species boundaries.

In this regard, delimiting species boundaries using ecological niche modeling (ENM) approach (alongside molecular studies) has generated wide interest [9], [10], [11], [12], [13], [14], [15], [16], [17]. Martinez et al, [18] observe that these ecological niche models, by identifying consistent differentiation patterns in characters related to the ecological niche, might provide alternative means of recognizing putatively independent lineages and thus act as an effective tool in delimiting species boundaries. These models which largely employ empirical data are useful to characterize species ecological requirements [19], [20], [21], understand distributions, biogeography and dispersal barriers [10], [22], identify effects of climate change [14], [23], forecast species invasions [24], realize the effects of habitat alterations [25], delimit species boundaries [9], [11], [12], [13] and predict unknown populations and species [26], [27].

Ecological niche models are being utilized for a number of aforementioned reasons, and its application in the field of Primatology also seems to be slowly gaining momentum. Primates play an important role in seed dispersal thus helping to maintain and balance biodiversity [28], [29], [30]. However, studies using primates as model systems largely assess the status and potential distributions for setting up conservation priorities [18], [26], [31], [32], [33], [34].

Of recognized 634 primates in the world, at least 304 of them are threatened with extinction [35]. It implies that nearly half of the world's primates are at risk. The primates as a whole are facing the worst odds in all the years they have been assessed and there are many more to follow [36]. However the number of “species” at risk of extinction still remains uncertain due to the ambiguities regarding the definition of species concepts itself indicating, that our understanding of primate diversity and taxonomy is by no means complete [37]. Taxonomy solely based on specific morphological traits often results in inadequate or misleading guides for phylogenetic distinctions at subspecies and species level [38], [39]. Hanuman langurs (*Semnopithecus entellus*) are one such example, whose taxonomic status is hugely debated.

Amongst the most widely distributed non-human primates in South Asia, Hanuman langurs or common langurs (Family: Cercopithecidae, Subfamily: Colobinae) are a common sight in Indian villages, towns and tourist areas, [40], [41]. They are also revered by Hindus, and perhaps one of the most extensively studied non-human diurnal primates in India. Hanuman langurs are distributed throughout most of India and Sri Lanka [42], [43], [44] as well as in parts of Pakistan, Nepal [43], [45], [46], Bhutan and Bangladesh [47]. Hanuman langurs are acclimatized to a wide range of habitats [48], [49], [50], [51], [52], [53], [54], [55], [56], [57] from arid regions on the edge of the desert in Rajasthan to the rainforests of Western Ghats. They have been recorded at altitudes from sea level to 4270m above msl in the Himalayas [58], [59], [60]. The annual rainfall in Hanuman langur habitats is known to range between 10 cm to 740 cm [30], [49], [50], [51], [53], [55], [56], [61]. Hanuman langurs are also known to show adaptation to strong seasonality from Himalayan habitats (−7°C) to extreme summer temperatures at Rajasthan ranging between 30°C to 46°C [57], [59], [62], [63]. Being predominately folivorous, the Hanuman langur's diet includes mature leaves of deciduous and evergreen trees along with fruits, fruit buds and petioles [30], [54], [57], [64], [65].

There has been much disagreement in the literature on the subspecies or species status of various populations of Hanuman langurs [58]. Most authors consider Hanuman langurs to be a single species (*Semnopithecus entellus*), but classify it into 14, 15, and 16 subspecies while others split them into two, four and seven distinct species suggesting their taxonomy is in a flux [66]. A recent work by Nag et al, [58] observed at least six morphotypes of Hanuman langurs in peninsular India by using a combination of five diagnostic morphological characters. Their study recommended Hill's [67] classification scheme for future studies to bring about some clarity in the taxonomy of these langurs. However, these results now need to be validated with other lines of evidence such as ecology and molecular data.

Hill [67] classified Hanuman langurs into four species namely *Semnopithecus schistaceus*, *S. entellus*, *S. hypoleucos* and *S. priam*. While the *S. schistaceus* and *S. entellus* have Northern type (NT) tail carriage distributed to the north of Narmada and Krishna rivers of peninsular India, *S. hypoleucos* and *S. priam* on the other hand have Southern type (ST) tail carriage and are predominantly distributed south of Narmada and Krishna rivers in South India and Sri Lanka. *Semnopithecus schistaceus* consists of five subspecies viz., *S. s. hector*, *S. s. schistaceus*, *S. s. achilles*, *S. s. ajax* and *S. s. lanius*, largely confined to the Himalayas. *S. entellus* is distributed in the plains of central and northern India (south of the Himalayan region) till the Narmada and Krishna rivers of peninsular India. The southern species *S. priam* consists of three subspecies namely *S. p. priam, S. p. thersites* and *S. p. anchises*; *S. hypoleucos* consists of six subspecies *S. h. hypoleucos, S. h. aeneas, S. h. elissa, S. h. iulus*, *S. h. dussumieri* and *S. h. achates*. However, Hill [67] was doubtful of the validity of *S. p. thersites* and *S. h. dussumieri* [58]. Thus one of the fundamental questions is that whether morphologically distinct species and subspecies of Hanuman langurs also exhibit distinct ecological niches?

The majority of Hanuman langur studies in India and Sri Lanka have directed their attention to behavioral studies (Nag, unpublished) and a few studies have looked at foraging ecology [30], [51], [54], [57], [68], [69], [70], [71], [72]. Furthermore, there has been a lack of a reliable distributional and ecological data on various species/subspecies of Hanuman langurs. Accordingly, there is an urgent need to delimit species and subspecies boundaries among Hanuman langurs and understand their requirements. This is particularly important given that the Hanuman langurs are used as model organisms for various biomedical, ecological, behavioral studies [66]. Thus in this paper, we test if the species and subspecies accepted by Hill [67] exhibit significant divergence in their ecological niches. Also, we have attempted to understand their ecological requirements and potential distributional ranges. In order to do so we concentrated on the southern species *S. priam* and *S. hypoleucos* and their subspecies. Furthermore we have also included *S. entellus* and *S. johnii* in the analysis.

## Materials and Methods

### Ethics statement

All the observations were made without any physical contact with the study animals. To carry out necessary field work we had necessary permissions from the Forest Departments of respective State Governments, which is a regulatory body (Andhra Pradesh State Rc. No. 29757/2009/WL.3 dated 24/8/2009; Kerala State WL.1 2-2937/2008 dated 26/7/2008; Karnataka State PCCF/C/CR-127/2007-2008 dated 20/5/2008 & D/WL/CR-/2007-2008 dated 23/1/2008 and Maharashtra state D-22(8)/Research/1340/2009-2010 dated 4th August/September 2009).

### Target species and occurrence data

**Figure 1:**
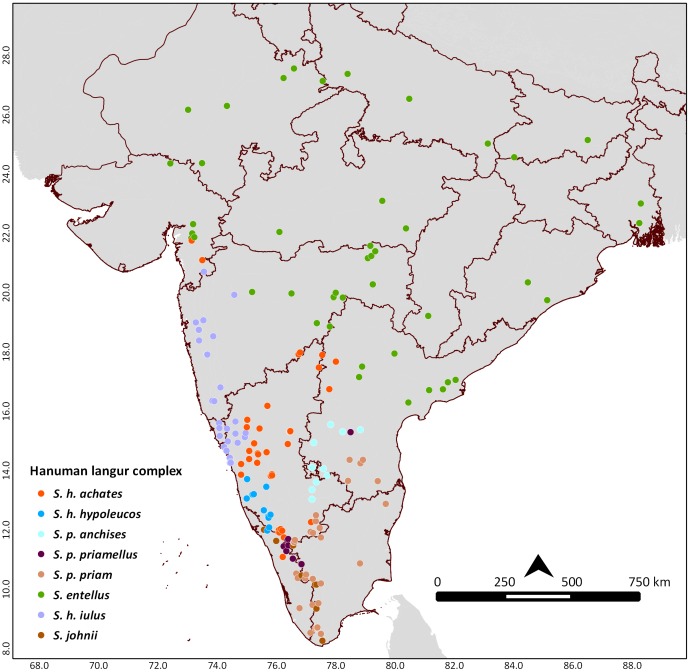
Occurrence points of eight taxa of langurs of peninsular India.

The study obtained 196 non-overlapping occurrence records of seven Hanuman langur morphotypes namely, Semnopithecus hypoleucos achates, S. h. iulus, S. h. hypoleucos, Semnopithecus priam priamellus, S. p. priam, S. p. anchises, S. entellus; and Nilgiri langur Semnopithecus johnii [58]. We followed Hill's [67] classification scheme to assign species/subspecies names for the morphotypes. We retained name priamellus as per Pocock, [73] for one population in Palakkad district. The occurrence records were collected from field surveys (details of field surveys are given in [58]) and literature records [74], [75] representing the known distribution of the species (Table 1; Figure 1). The occurrence data are available with the corresponding author and will be sent on request.

**Table 1:** Non-overlapping occurrence data points of the various taxa used in the present study.

Sl. No.	Species/Subspecies	Field survey data	Secondary data	Total
**1**	*S. hypoleucos achates*	32	8	40
**2**	*S. hypoleucos hypoleucos*	7	5	12
**3**	*S. priam anchises*	10	2	12
**4**	*S. priam priamellus*	10	1	11
**5**	*S. priam priam*	22	13	35
**6**	*S. entellus*	21	24	45
**7**	*S. hypoleucos iulus*	26	4	30
**8**	*S. johnii*	6	5	11
**Total occurrence points**				**196**

### Environmental Coverage Variables

The study considered 37 environmental variables for modeling ecological niches of Hanuman langurs in peninsular India. Of these 37 variables, 19 were bioclimatic [76], 12 were vegetation (Enhanced Vegetation Index – EVI) and 6 were hydrological layers. The layers were related to precipitation, temperature, topography, and ecological bioregions (Table S1 in File S1). All the layers were re-sampled to 1000 m resolution, on WGS84 Longitude-Latitude projection and clipped for Indian subcontinent (excluding Sri Lanka, Bangladesh, Pakistan, Nepal and Bhutan). Layers were tested for multicollinearity and layers that had r≤±0.85 (Pearson's correlation coefficient) following Elith et al, [77] were selected for further analysis. This resulted in 14 layers comprising of one vegetation layer, seven bioclimatic and six hydrological layers.

### MaxEnt modeling algorithm

We used a maximum entropy algorithm available in MaxEnt [78], [79]. Recent studies indicate [17], [80], [81] that MaxEnt performs well when compared with other ENM methods and has been widely used to delimit species boundaries and ecological niches [82], [83], [84], [85]. MaxEnt is a machine learning program that estimates the probability distribution for a species occurrence based on environmental constraints [79]. MaxEnt is designed to make predictions from presence-only data using background environment of the study area. In other words MaxEnt is designed to characterize probability distribution from incomplete information. MaxEnt is also advantageous since it uses both continuous and categorical variables [86] and the output is a continuous prediction. MaxEnt has been in wide use because of its effectiveness even with small sample sizes. However, few drawbacks of MaxEnt approach such as model extrapolation or over fitting have been discussed in the literature [86].

MaxEnt was used with following changes in the model run. Random test percentage was set to 25%. Regularization multiplier was set to 1 and maximum number of background points for sampling was kept at 10,000. Extent of background selection points was restricted to the regions of mutually exclusive species occurrence points drawing minimum convex polygons using QGIS. We generated eight separate polygons. Within these polygons 10,000 random background points were selected. As species occurrence points used in the study was not collected randomly, we provided bias files for each species separately during MaxEnt modeling. Each bias grid file is generated in QGIS applying Gaussian kernel function to 10,000 background points following Elith et al, [77]. We ran 15 replicates for each species and averaged the results. Maximum iterations were set to 5000, with 1*10^−6^ as convergence threshold. Auto feature of environmental variables was selected. A 15 fold cross-validation was used to test model performance of each species. Jackknife procedure and percent variable contributions were used to estimate the environmental variable influence on each species. We performed Correspondence analysis (CA), an ordination analysis on each species with their respective percentage contribution of the environmental variables. Correspondence analysis use Chi square distance (χ^2^) to quantify the relationships among the dimensionally homogenous data set [87].

Logistic modeling output was chosen that displays suitability values from 0 (unsuitable) to 1 (optimal). For extracting the range values of environmental variables from logistic output, we considered threshold values ≥0.75 for all species following Liu et al, [88]. We derived threshold value from least valued ROC (Receiver Operator Characteristic) plot (Average ROC is 0.894±0.02 for *S. entellus*), wherein the threshold point (north westernmost point) lies at the intersection of the ROC curve and the line perpendicular to the diagonal of no discrimination following Lobo et al, [89].

The logistic model output is converted to binomial output with ‘0’ to values from 0–0.75 and ‘1’ to values ≥0.75. Area under curve (AUC) value is calculated for model validation. AUC reflects the model's ability to distinguish between presence records and random background points. AUC values ranged from 0.5 (not different from a randomly selected predictive distribution) to 1 (with perfect predictive ability). Models having AUC values >0.9 were considered to have very good, >0.8 good and >0.7 useful discrimination abilities [90]. We calculated partial receiver operator characteristics (pROC) as an additional measure to check model performances following Peterson et al, [91]. We used pROC calculator developed by Barve [92]. Z test was performed for statistical significance of the pROC values. Spatial overlaps between two species are calculated using QGIS and Idrisi® Taiga.

Using ENMTools software, niche overlap was measured among species distribution. Schoener's D as a measure of niche overlap [93] was estimated by taking the difference between species in suitability score at each grid cell, after suitabilities were standardized [94], [95], [96]. This metric ranged from 0 (species without any niche overlap) to 1 (species with complete niche overlap/identical niches). D values >0.8 was considered as significant niche overlap [94].

## Results

### Species distribution

**Figure 2:**
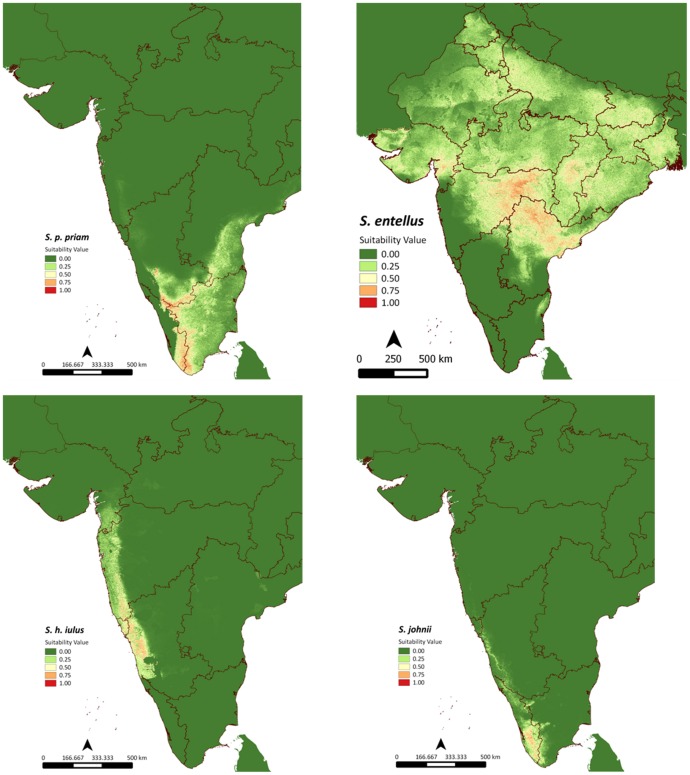
Maxent distribution modeling logistic output for *S. p. priam*, *S. entellus*, *S. h. iulus* and *S. johnii*.

**Figure 3:**
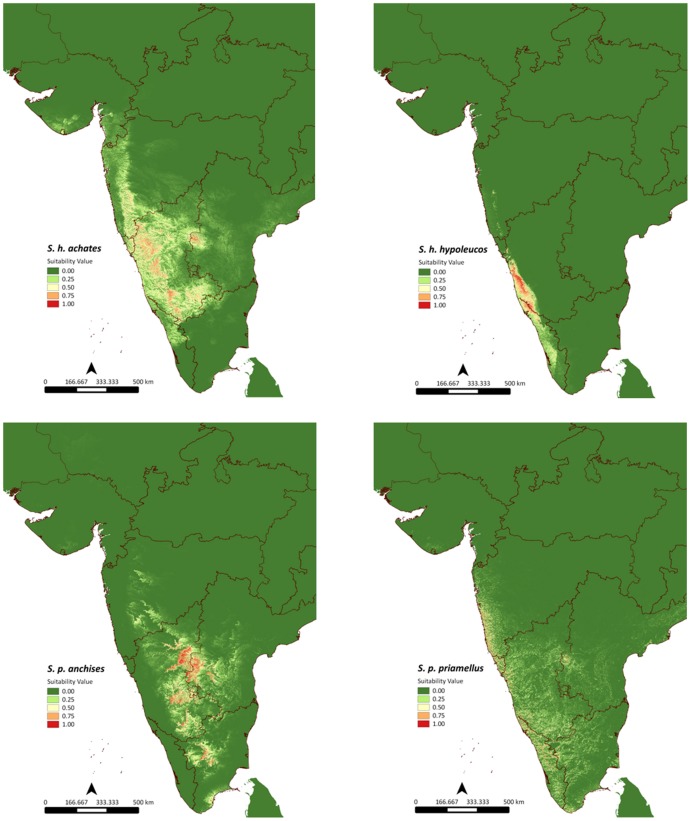
Maxent distribution modeling logistic output for *S. h. achates, S. h. hypoleucos, S. p. anchises* and *S. p. priamellus*.

Based on maximum entropy modeling algorithm and using 14 environmental variables, we obtained eight distinct distribution maps corresponding to *S. h. achates, S. h. iulus, S. h. hypoleucos, S. p. priamellus, S. p. priam, S. p. anchises, S. entellus* and *S. johnii*. Figure 2 and 3 shows the distribution maps with warmer colours indicating more suitable habitat and cooler colours indicating unsuitable habitats.

### Model prediction and assessment

Area of each species in predicted distribution is given in Table 2. Total area predicted is 49,95,934 km^2^. *Semnopethicus entellus* has the highest area under the prediction (28,34,717 km^2^) while it was least in *S. h. hypoleucos* (91,722 km^2^). There was no significance correlation between the number of data points used for modeling and the area predicted (r = 0.65, p = 0.079). Percent suitability area predicted was highest in *S. h. hypoleucos* with 6.2% of total area predicted, followed by *S. p. anchises* with 3.18% and was least in *S. h. iulus* with 0.55%.

**Table 2:** Predicted area of distribution for all the taxa.

Species	Predicted area (km^2^)	Area ≥0.75 suitability (km^2^)	% of total area
***S. h. achates***	620559	4244	0.68
***S. h. hypoleucos***	91722	5695	6.2
***S. p. anchises***	367276	11660	3.18
***S. p. priamellus***	377501	3569	0.95
***S. p. priam***	380746	4736	1.24
***S. entellus***	2834717	18832	0.66
***S. h. iulus***	201720	1104	0.55
***S. johnii***	121693	1378	1.13

All the model performances exhibited high mean AUC values for 15 replicates in each species (Range: 0.894–0.989) (Table 3). The partial ROC values for each species was well over 1.0 (Range: 1.015–1.981) and were statistically significant (Z test, p<0.0001).

**Table 3:** Area Under Curve (AUC) and Partial ROC values.

Species	AUC±SD	Partial ROC±SD
***S. h. achates***	0.942±0.051	1.814±0.129*
***S. h. hypoleucos***	0.948±0.105	1.520±0.372*
***S. p. anchises***	0.907±0.125	1.513±0.346*
***S. p. priamellus***	0.960±0.081	1.466±0.369*
***S. p. priam***	0.989±0.003	1.933±0.006*
***S. entellus***	0.894±0.02	1.485±0.063*
***S. h. iulus***	0.982±0.022	1.389±0.282*
***S. johnii***	0.967±0.077	1.986±0.002*

Note: * indicates Z test significance at P<0.0001.

### Variable range and their importance

**Figure 4:**
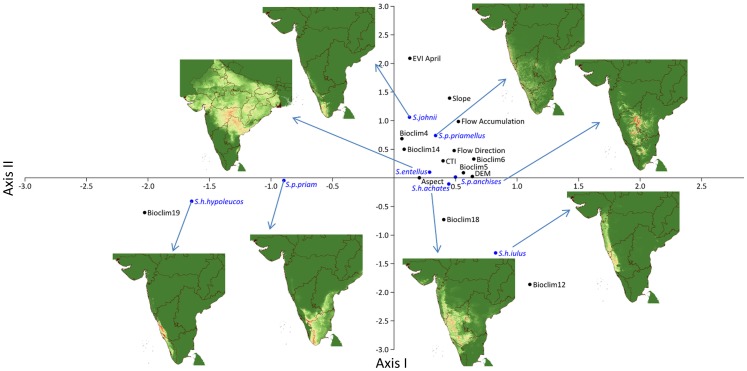
Correspondence analysis of variables of importance based from MaxEnt modeling output.

Variables and permutation importance for each species are given in Table S2 in File S1. In addition the range of environmental variables in the predicted regions is given in Table S3 in File S1. Considering the importance values of ecological variables of each species, we performed correspondence analysis (Figure 4). Axis I and II explains 30% and 22% of variations in the data sets (Table S4 in File S1). Figures S1–S8 in File S1 provide response curve plots that show the type of correlation and influence of predicted suitability on the environmental variables (top five variables with high percentage contribution).

Temperature seasonality (Bioclim4) was primary variable influencing the niche of *S. p. anchises* and *S. p. priamellus* (25.4% and 30.3% respectively) niches. Similarly, it was precipitation during coldest quarter (Bioclim19) in *S. h. hypoleucos* and *S. p. priam* niches (82.7% and 48.5% respectively). Annual precipitation (Bioclim12) in *S. h. iulus* niche (73.6%). In *S. h. achates* and *S. entellus* niches it was maximum temperature in warmest month (Bioclim5) which had highest contribution (24.9% and 45.6% respectively). Precipitation during the driest month (Bioclim14) influence *S. johnii*'s niche (38.2%). Vegetation index of April was second important variable in *S. johnii*'s niche (33.1%). Among the hydrological parameters, digital elevation model, slope, aspect, flow direction and flow accumulation had significant contribution in determining ecological niches (Table S2 in File S1).

### Niche overlap

Table 4, provides percentage niche overlap between species pairs. Percentage overlap ranged from 0 to 2.82%, with most species pairs showing zero overlap. Table 5, illustrates the pair wise niche overlap by each taxa. Here again none of the taxa pairs exhibited values beyond 8%, which clearly indicates distinct niches for each taxa. For an independent measure of modeled output, we measured D statistic values. None of the niche overlap estimate using D statistics showed any significant overlap (Table 6).

**Table 4:** Percentage Niche overlaps (%) between taxa pairs to their area of prediction.

Species	*S.h. hypoleucos*	*S.p. anchises*	*S.p. priamellus*	*S.p. priam*	*S. entellus*	*S.h. iulus*	*S. johnii*
***S.h. achates***	0.01	2.18	0.82	0.02	0.00	0.00	0.00
***S.h.hypoleucos***		0.00	2.82	0.00	0.00	0.74	0.01
***S.p.anchises***			1.14	0.01	0.00	0.00	0.00
***S.p.priamellus***				0.30	0.00	0.47	0.10
***S.p.priam***					0.00	0.00	0.47
***S. entellus***						0.00	0.00
***S.h.iulus***							0.00

**Table 5:** Proportion of the predicted area of taxa A occupied by the predicted area of taxa B.

Species	*S. h. achates*	*S. h.* *hypoleucos*	*S. p. anchises*	*S. p. priamellus*	*S. p. priam*	*S. entellus*	*S. h. iulus*	*S. johnii*
***S.h.achates***	-	0.02	8.18	1.51	0.05	0.00	0.00	0.00
***S.h.hypoleucas***	0.02	-	0.00	4.58	0.00	0.00	0.88	0.02
***S.p.anchises***	2.98	0.00	-	1.48	0.01	0.00	0.00	0.00
***S.p.priamellus***	1.79	7.31	4.85	-	0.70	0.00	0.62	0.14
***S.p.priam***	0.04	0.00	0.02	0.53	-	0.00	0.13	0.61
***S. entellus***	0.00	0.00	0.00	0.00	0.00	-	0.00	0.00
***S.h. iulus***	0.00	4.53	0.00	1.99	0.54	0.00	-	0.00
***S. johnii***	0.00	0.07	0.00	0.36	2.10	0.00	0.00	-

**For above the diagonal values, species in row-heads are taxa A and column-heads are taxa B. For below the diagonal values, taxa in row-heads are taxa B and column-heads are taxa A.**

**Table 6:** Niche overlap estimate (D statistic) between species pairs.

Species	*S. h. hypoleucos*	*S. p. anchises*	*S. p. priamellus*	*S. p. priam*	*S. entellus*	*S. h. iulus*	*S. johnii*
***S. h. achates***	0.124	0.294	0.364	0.157	0.066	0.309	0.100
***S. h. hypoleucos***		0.205	0.336	0.061	0.002	0.171	0.435
***S. p. anchises***			0.381	0.150	0.029	0.028	0.244
***S. p. priamellus***				0.230	0.028	0.217	0.311
***S. p. priam***					0.020	0.023	0.244
***S. entellus***						0.032	0.001
***S. h. iulus***							0.064

## Discussion

Ecological niche modeling based on maximum entropy (MaxEnt) algorithm was used to determine the distinct ecological niches of various taxa of langurs of peninsular According to Hill [67], there are three species of Hanuman langurs in South India viz., *S. entellus; S. hypoleucos* and *S. priam* and a sister taxa Nilgiri langur (*S. johnii*). According to Nag et al, [58], based on morphology there are three subspecies in *S. hypoleucos* and three species in *S. priam*, making a total of eight taxa of langurs in peninsular India. In the present study, ENM clearly demarcated the ecological niches of these taxa mentioned above, with significant AUC and pROC in each of the distribution model. Selection of a threshold value to convert predicted model layer to binary layer has a significant influence on model accuracy, especially for presence-only data set [78], [88]. With increase in threshold values there will be a decrease in predicted suitable area of the species. This has further implications on biodiversity assessment, protected/reserve area selection, climate change impact studies and government policies on conservation of a species [89], [97]. For a sound reserve design and conservation programme on Hanuman langur complex one needs to carefully look at the threshold values used in binary model prediction layer.

Bioclim variables had a major contribution in determining the niche of a particular species followed by hydrological layers and EVI (Figure 4). It is well known that *S. johnii* inhabits evergreen forests of high elevation in south-west India between 8°–10°N latitude and the region receives rainfall throughout the year [58]. However, it is the precipitation in driest month and vegetation index of April predominantly influenced ecological niche of *S. johnii*.

Similarly, the niche of *S. h. hypoleucos* is determined predominantly by precipitation during the coldest quarter (82.7%) in the mid Western Ghats regions of south-west India between 12°–14°N. Ecological niche of *S. h. iulus* was between 14°–18°N on the Western part of south-west India predominantly determined by annual precipitation (73.6%). This particular region receives moderate rainfall [72].

In the present study, the distribution of *S. p. priam* was between 8°–13°N and strongly influenced by precipitation during coldest quarter. *S. p. priam* is confined to deciduous forests in southern Western Ghats of India [58]. For the most widely distributed taxa in India, *S. entellus*, it is the temperature during warmest month and temperature seasonality that determined the niche, followed by aspect. Earlier studies have indicated an influence of temperature on vegetation type [63], [97].

Niche of *S. h. achates* is between 12°–17°N and 76°–79°E influenced by maximum temperature in warmest month followed by annual precipitation. This corroborated with the fact that *S. h. achates* occured in the regions with very low rainfall and dry vegetation [48], [58]. Niche of *S. p. anchises* is influenced by temperature seasonality in the south-central India between 11°–17°N and 77°–79°E. It is also the taxa belonging to low rainfall and dry vegetation region [58]. Ecological niche of *S. p. priamellus* was also determined by temperature seasonality, warranting more studies.

Through ENM, the study looked at niche overlaps between taxa pairs to determine if there was divergence in their ecological axis. There were no significant niche overlaps between any taxa pairs, thereby suggesting that each taxa occupied a distinct ecological niche. This clearly supported the morphological distinction of Hanuman langurs as explained in Nag et al, [58]. *Semnopithecus johnii* has long been considered as a distinct species and can be easily distinguished from the members of the Hanuman langur species complex by their distinct pelage color and vocalizations [75], [98], [99]. They have a very restricted distribution and are confined to the wet evergreen forest of Central and Southern Western Ghats.Nevertheless, in some areas mixed species associations between *S. johnii* and *S. priam* have been reported [52], [56], [98], [99] and sometimes they are also known to hybridize. Such polyspecific association and hybridization events have been reported for other Asian primates as well [51], [68], [69], [100], [101], [102], [103]. Nonetheless, ecological niche modeling provided a distinct, non-overlapping niche for *S. johnii* and supported it as a distinct species. Another interesting output of the model was the support for Pocock's [73] *priamellus* which has a very restricted distribution in Western Ghats. According to Nag et al, [58], *S. p. priamellus* form is morphologically distinct from other morphotypes of *Semnopithecus* and is confined to Nilambur, Silent valley national park and Walayar regions of Northern Kerala. Interestingly, Hill's classification scheme discussed *priamellus* specimens as “doubtful” and he subsumed it under *S. priam*. However our analysis shows that its niche is distinct from all other taxa with very little overlap. Thus our results provide reasonably good support for retaining *priamellus* as a valid taxa as a subspecies of *S. priam*. However, a detailed survey of this population should be undertaken to better understand its distributional limits.

Although this exercise has provided greater clarity regarding niche separation between members of the Hanuman langur complex in south India, there are limitations and uncertainties in the modeling process, occurrence data and layers used in such studies as indicated in similar modeling studies elsewhere [104]. We used MaxEnt software for this study considering the use and performance [82], [83], [84], [85], however there is no single best algorithm or software that addresses the uncertainties of modeling process [17]. A platform for ensemble forecasting called BIOMOD [23] is proposed to overcome the limitations of single model predictions. Also, small occurrence data (there were four species with <12 occurrence data) increase the uncertainty of predicting the ecological niches [105]. We cross validated individual model run to overcome this uncertainty.

Our observations in the present study should be cautiously weighed in the light of limitations of ENM, in that using only spatial variables may not give us complete niche separation of species. Thus, one has to look at other variables of species, *viz.*, canopy density, habitat preference, breeding behavior, feeding pattern, troop dynamics, and niche occupancy which can provide much deeper insight on realized niches of each species. Adding these variables might generate more precise species boundaries.

## Supporting Information

File S1 **Combined supporting information file containing Tables S1–S4, Figures S1–S8.** Table S1. Derived bioclimatic, hydrological and vegetation layers used in the present study. Each layer is with 1000 m resolution and is clipped for Indian sub-continent. Table S2. Variables with percent contribution (in bold) and permutation importance in predicted distribution of species. Table S3. Range of Environmental variables in the predicted suitability regions. Table S4. Eigenvalue and percentage variation explained in correspondence analysis. Figure S1. Response curves of top five variables of importance in *S. h. achates*. Figure S2. Response curves of top five variables of importance in *S. h. hypoleucos*. Figure S3. Response curves of top five variables of importance in *S. p. anchises*. Figure S4. Response curves of top five variables of importance in *S. p. priamellus*. Figure S5. Response curves of top five variables of importance in *S. p. priam*. Figure S6. Response curves of top five variables of importance in *S. entellus*. Figure S7. Response curves of top five variables of importance *S. h. iulus*. Figure S8. Response curves of top five variables of importance in *S. johnii*.
(DOC)
