# Correction: Delineating Ecological Boundaries of Hanuman Langur Species Complex in Peninsular India Using MaxEnt Modeling Approach

**DOI:** 10.1371/journal.pone.0091497

**Published:** 2014-02-28

**Authors:** The PLOS ONE Staff

The names of all authors are incorrectly represented in the Citation. The correct Citation is: Nag C, Karanth KP, Gururaja KV (2014) Delineating Ecological Boundaries of Hanuman Langur Species Complex in Peninsular India Using MaxEnt Modeling Approach. PLoS ONE 9(2): e87804. doi:10.1371/journal.pone.0087804.
