# Supplementary material for: Delineating Ecological Boundaries of Hanuman Langur Species Complex in Peninsular India Using MaxEnt Modeling Approach
Source: PLoS One. 2014 Feb 3;9(2):e87804. doi: 10.1371/journal.pone.0087804 (PMC3912124; doi:10.1371/journal.pone.0087804)

Table S1. Derived bioclimatic, hydrological and vegetation layers used in the present study. Each layer is with 1000m resolution and is clipped for Indian sub-continent.

| **Layer** | **Variable** | | **Reference** | |
| --- | --- | --- | --- | --- |
| **Bioclimatic** |  | |  | |
| Bioclim1 | Annual Mean Temperature (°C*10) | | [http://www.worldclim.org](http://www.worldclim.org/) [76] | |
| Bioclim2 | MeanDiurnalRange (Mean (period max-min)) (°C*10) | | [http://www.worldclim.org](http://www.worldclim.org/) [76] | |
| Bioclim3 | Isothermality (Bioclim2/Bioclim7) (°C*10) | | [http://www.worldclim.org](http://www.worldclim.org/) [76] | |
| Bioclim4 | Temperature Seasonality (SD*100) | | [http://www.worldclim.org](http://www.worldclim.org/) [76] | |
| Bioclim5 | Max Temperature of Warmest month (°C*10) | | [http://www.worldclim.org](http://www.worldclim.org/) [76] | |
| Bioclim6 | Min Temperature of Coldest month (°C*10) | | [http://www.worldclim.org](http://www.worldclim.org/) [76] | |
| Bioclim7 | TemperatureAnnualRange (Bioclim5-Bioclim6) | | [http://www.worldclim.org](http://www.worldclim.org/) [76] | |
| Bioclim8 | Mean Temperature of Wettest Quarter (°C*10) | | [http://www.worldclim.org](http://www.worldclim.org/) [76] | |
| Bioclim9 | Mean Temperature of Driest Quarter (°C*10) | | [http://www.worldclim.org](http://www.worldclim.org/) [76] | |
| Bioclim10 | Mean Temperature of Warmest Quarter (°C*10) | | [http://www.worldclim.org](http://www.worldclim.org/) [76] | |
| Bioclim11 | Mean Temperature of Coldest Quarter (°C*10) | | [http://www.worldclim.org](http://www.worldclim.org/) [76] | |
| Bioclim12 | Annual Precipitation (mm) | | [http://www.worldclim.org](http://www.worldclim.org/) [76] | |
| Bioclim13 | Precipitation of Wettest Period (mm) | | [http://www.worldclim.org](http://www.worldclim.org/) [76] | |
| Bioclim14 | Precipitation of Driest Period (mm) | | [http://www.worldclim.org](http://www.worldclim.org/) [76] | |
| Bioclim15 | Precipitation Seasonality (Coefficient of Variation) | | [http://www.worldclim.org](http://www.worldclim.org/) [76] | |
| Bioclim16 | Precipitation of Wettest Quarter (mm) | | [http://www.worldclim.org](http://www.worldclim.org/) [76] | |
| Bioclim17 | Precipitation of Driest Quarter (mm) | | [http://www.worldclim.org](http://www.worldclim.org/) [76] | |
| Bioclim18 | Precipitation of Warmest Quarter (mm) | | [http://www.worldclim.org](http://www.worldclim.org/) [76] | |
| Bioclim19 | Precipitation of Coldest Quarter (mm) | | [http://www.worldclim.org](http://www.worldclim.org/) [76] | |
| **Hydrological** |  | | |  |
| DEM | Digital Elevation model | | | USGS, EROS centre, Hydro1k for Asia |
| Aspect | Direction of slope | | | USGS, EROS centre, Hydro1k for Asia |
| Slope | Difference between two neighboring cells elevation | | | USGS, EROS centre, Hydro1k for Asia |
| CTI | Composite Topographic Index (Wetness Index) | | | USGS, EROS centre, Hydro1k for Asia |
| FA | Flow accumulation | | | USGS, EROS centre, Hydro1k for Asia |
| FD | Flow direction | | | USGS, EROS centre, Hydro1k for Asia |
| **Enhanced Vegetation Index (EVI)** | | | | |
| EVI_January_2001 | | MODIS, Global Land Cover Facility, <http://glcf.umiacs.umd.edu/index.shtml> | | |
| EVI_February_2001 | | MODIS, Global Land Cover Facility, <http://glcf.umiacs.umd.edu/index.shtml> | | |
| EVI_March_2001 | | MODIS, Global Land Cover Facility, <http://glcf.umiacs.umd.edu/index.shtml> | | |
| EVI_April_2001 | | MODIS, Global Land Cover Facility, <http://glcf.umiacs.umd.edu/index.shtml> | | |
| EVI_May_2001 | | MODIS, Global Land Cover Facility, <http://glcf.umiacs.umd.edu/index.shtml> | | |
| EVI_June_2001 | | MODIS, Global Land Cover Facility, <http://glcf.umiacs.umd.edu/index.shtml> | | |
| EVI_July_2001 | | MODIS, Global Land Cover Facility, <http://glcf.umiacs.umd.edu/index.shtml> | | |
| EVI_August_2001 | | MODIS, Global Land Cover Facility, <http://glcf.umiacs.umd.edu/index.shtml> | | |
| EVI_September_2001 | | MODIS, Global Land Cover Facility, <http://glcf.umiacs.umd.edu/index.shtml> | | |
| EVI_October_2001 | | MODIS, Global Land Cover Facility, <http://glcf.umiacs.umd.edu/index.shtml> | | |
| EVI_November_2001 | | MODIS, Global Land Cover Facility, <http://glcf.umiacs.umd.edu/index.shtml> | | |
| EVI_December_2001 | | MODIS, Global Land Cover Facility, <http://glcf.umiacs.umd.edu/index.shtml> | | |

Table S2. Variables with percent contribution (in bold) and permutation importance in predicted distribution of species.

| Variable | *S.h. achates* | *S.h. hypoleucos* | *S.p. anchises* | *S.p. priamellus* | *S.p. priam* | *S. entellus* | *S.h. iulus* | *S. johnii* |
| --- | --- | --- | --- | --- | --- | --- | --- | --- |
| April EVI | **0.6**  0 | **0.9**  1.4 | **1.3**  6.8 | **0.1**  0 | **0.9**  0 | **0.1**  0.5 | **0**  0 | **33.1**  28.7 |
| Bioclim4 | **10.4**  28.1 | **9.6**  74.6 | **25.4**  9.3 | **30.3**  41.2 | **16.7**  19.6 | **18.3**  44.5 | **0.3**  9.8 | **21.2**  64.8 |
| Bioclim5 | **24.9**  13.6 | **0.1**  1.9 | **5.9**  15.4 | **1.7**  7.6 | **0.8**  1.1 | **45.6**  4.4 | **0.5**  0 | **0.9**  0.6 |
| Bioclim6 | **0.5**  0.1 | **0**  0 | **18.1**  28.7 | **6.5**  23.5 | **1.4**  0.1 | **0.8**  3.9 | **0.7**  1 | **0.1**  0 |
| Bioclim12 | **19**  42 | **0**  0 | **17.8**  11.9 | **1**  0.1 | **1.7**  0.1 | **2.5**  9.7 | **73.6**  43.3 | **0.7**  0 |
| Bioclim14 | **13.9**  5.8 | **0**  0 | **6.5**  6.9 | **1.8**  0.9 | **24.1**  0.9 | **3.9**  1.5 | **12.8**  32.6 | **38.2**  4 |
| Bioclim18 | **0.4**  0.2 | **1.1**  0.5 | **1**  0.8 | **0.4**  0.1 | **0.6**  0.2 | **5.8**  10.7 | **3.7**  5.6 | **0.2**  0 |
| Bioclim19 | **2.1**  1 | **82.7**  14.3 | **0**  0.5 | **1.1**  0 | **48.5**  76.3 | **5.1**  10 | **2.5**  0.2 | **0**  0 |
| Aspect | **9.6**  4.5 | **2.4**  2.2 | **1.3**  0.3 | **0.4**  0 | **1.2**  0.2 | **9.7**  9.3 | **0.3**  0.2 | **1.2**  0.6 |
| Slope | **0.6**  0.2 | **0.5**  3.5 | **0.4**  0 | **24.6**  3.7 | **0.8**  0.3 | **1.2**  1 | **0.5**  0.1 | **1.3**  0.3 |
| CTI | **0.7**  0.4 | **0.1**  1.4 | **5.4**  0.7 | **3.3**  2.7 | **1.8**  0.8 | **1.7**  1.1 | **0.5**  0.1 | **0**  0 |
| DEM | **2.4**  0.4 | **0.5**  0.1 | **10.9**  10.7 | **3.7**  1 | **0.5**  0.3 | **4.1**  1.1 | **1.9**  7 | **0**  0 |
| FA | **0.5**  0.7 | **0**  0 | **0.4**  0.3 | **10.4**  5.7 | **0.8**  0.1 | **0.1**  0.1 | **2.1**  0.1 | **2.4**  1 |
| FD | **14.4**  3 | **2**  0 | **5.6**  7.7 | **14.6**  13.4 | **0.2**  0.1 | **1.1**  2.2 | **0.6**  0.1 | **0.6**  0 |

Table S3. Range of Environmental variables in the predicted suitability regions.

| **Variables** | ***S.h. achates*** | ***S.h. hypoleucos*** | ***S.p. anchises*** | ***S.p. priamellus*** | ***S.p. priam*** | ***S. entellus*** | ***S.h. iulus*** | ***S.johnii*** |
| --- | --- | --- | --- | --- | --- | --- | --- | --- |
| **April EVI** | -3000-5630 | 0-6319 | -68-6212 | -3000-6415 | 0-6843 | -3000-6133 | 0-5833 | 0-7775 |
| **Bioclim4** | 0-2278 | 0-2127 | 0-3067 | 0-2225 | 0-2848 | 0-6616 | 0-2168 | 0-1200 |
| **Bioclim5** | 0-377 | 0-348 | 0-399 | 0-374 | 0-367 | 0-432 | 0-355 | 0-343 |
| **Bioclim6** | 0-218 | 0-222 | 0-225 | 0-236 | 0-229 | 0-198 | 0-203 | 0-245 |
| **Bioclim12** | 0-4165 | 0-7212 | 0-764 | 0-5234 | 0-3578 | 0-1499 | 0-6412 | 0-4072 |
| **Bioclim14** | 0-3 | 0-22 | 0-10 | 0-27 | 0-48 | 0-12 | 0 | 0-79 |
| **Bioclim18** | 0-316 | 0-586 | 0-211 | 0-1062 | 0-935 | 0-394 | 0-287 | 0-963 |
| **Bioclim19** | 0-3065 | 0-5162 | 0-336 | 0-3606 | 0-366 | 0-84 | 0-4808 | 0-2011 |
| **Aspect** | 0-251 | 0-255 | 0-255 | 0-255 | 0-255 | 0-255 | 0-255 | 0-255 |
| **Slope** | 0-226 | 0-255 | 0-254 | 0-255 | 0-255 | 0-217 | 0-241 | 0-255 |
| **CTI** | 0-255 | 0-255 | 0-255 | 0-255 | 0-255 | 0-255 | 0-255 | 0-255 |
| **DEM** | 0-255 | 0-255 | 0-255 | 0-255 | 0-255 | 0-246 | 0-170 | 0-254 |
| **FA** | 0-255 | 0-255 | 0-255 | 0-255 | 0-254 | 0-255 | 0-238 | 0-251 |
| **FD** | 0-32 | 0-128 | 0-64 | 0-32 | 0-241 | 0-128 | 0-64 | 0-241 |

Table S4. Eigenvalue and percentage variation explained in correspondence analysis.

| **Axis** | **Eigen value** | **Percent variance explained** | **Cumulative variance (%)** |
| --- | --- | --- | --- |
| 1 | 0.611 | 30.151 | 30.151 |
| 2 | 0.448 | 22.268 | 52.418 |
| 3 | 0.410 | 20.404 | 72.823 |
| 4 | 0.333 | 16.555 | 89.378 |
| 5 | 0.141 | 6.998 | 96.376 |
| 6 | 0.046 | 2.299 | 98.675 |
| 7 | 0.027 | 1.325 | 100 |

Figure S1. Response curves of top five variables of importance in *S. h. achates.*


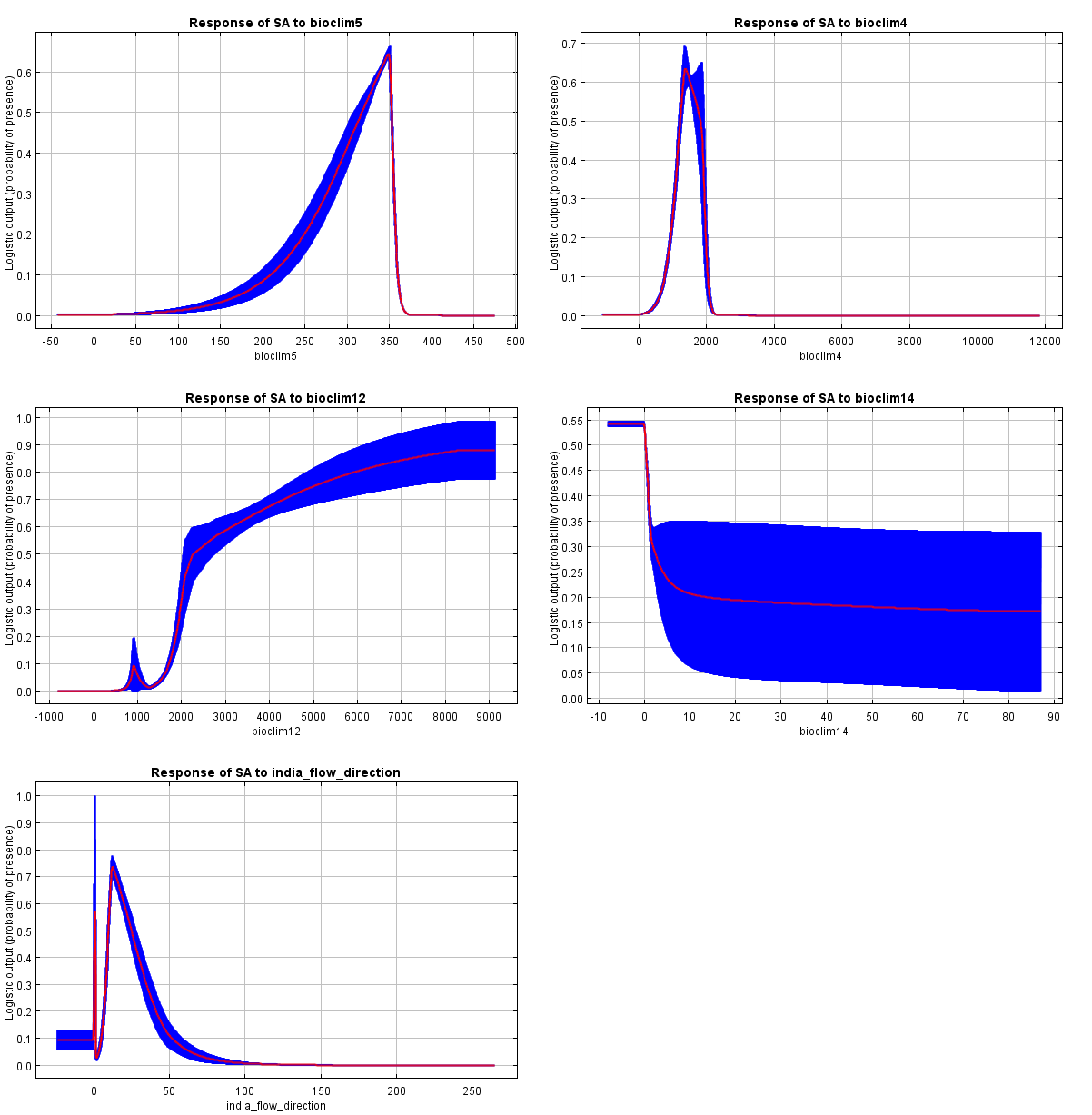


Figure S2. Response curves of top five variables of importance in *S. h. hypoleucos*.


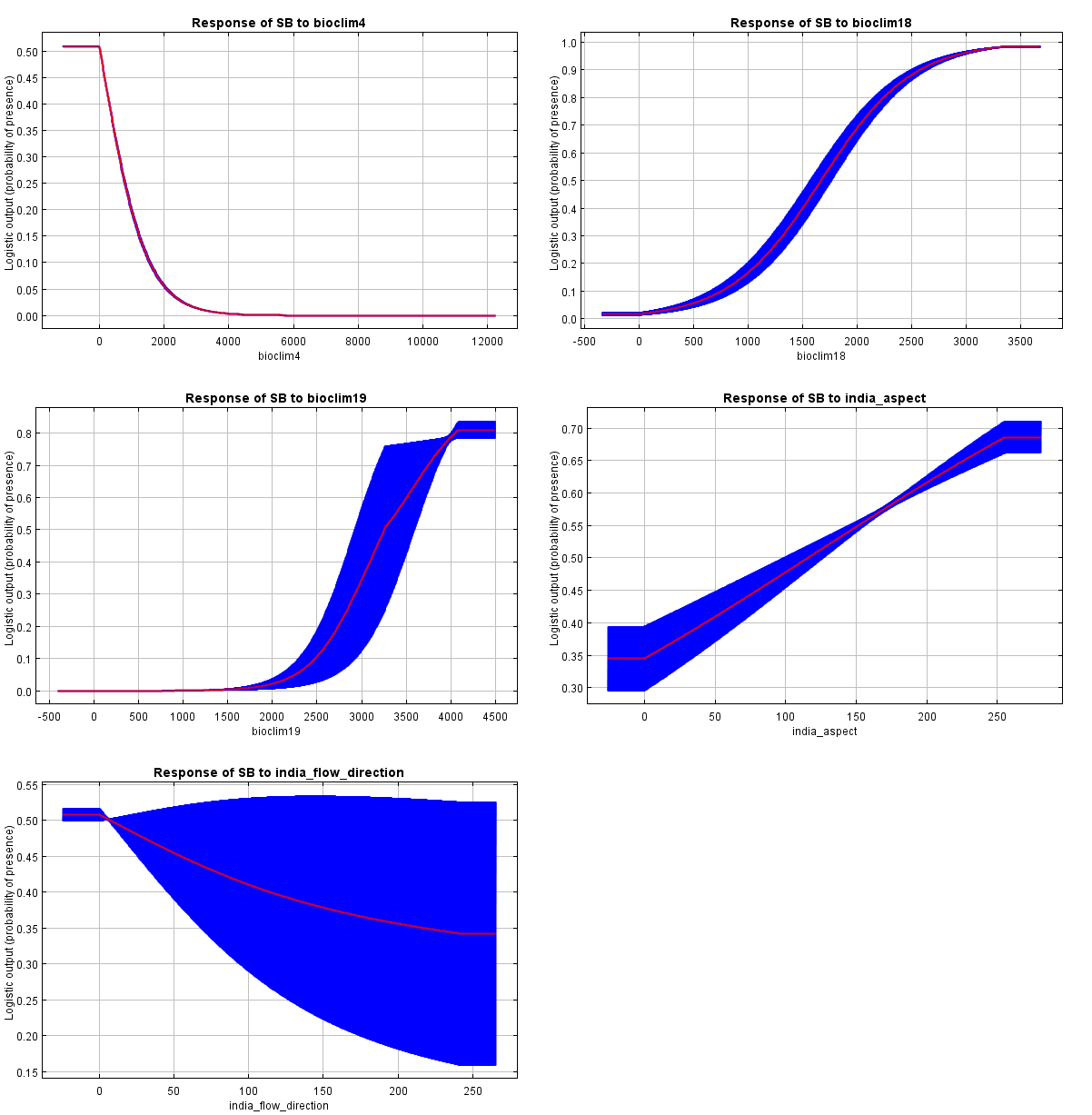


Figure S3. Response curves of top five variables of importance in *S. p. anchises*.


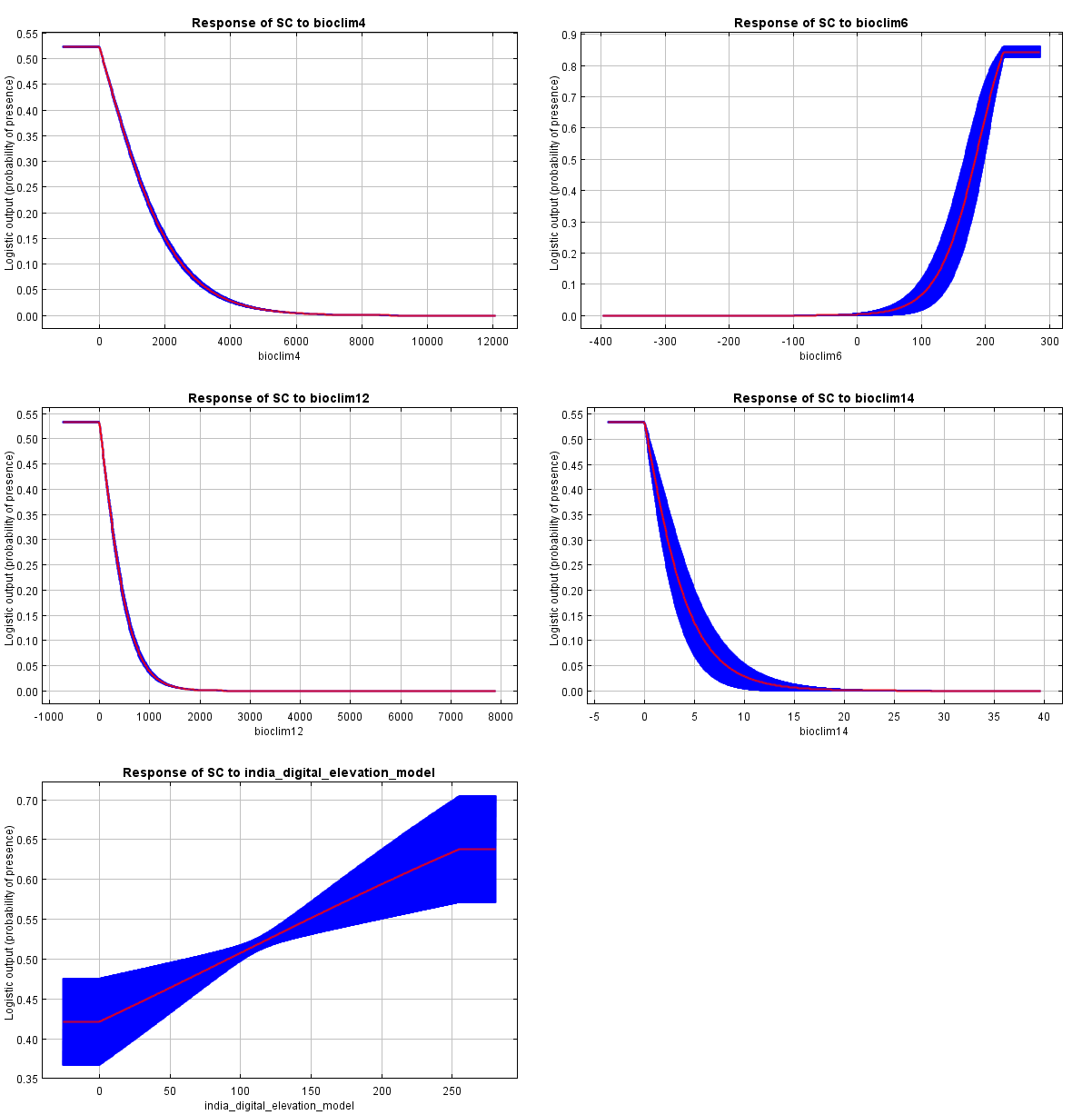


Figure S4. Response curves of top five variables of importance in *S. p. priamellus*.


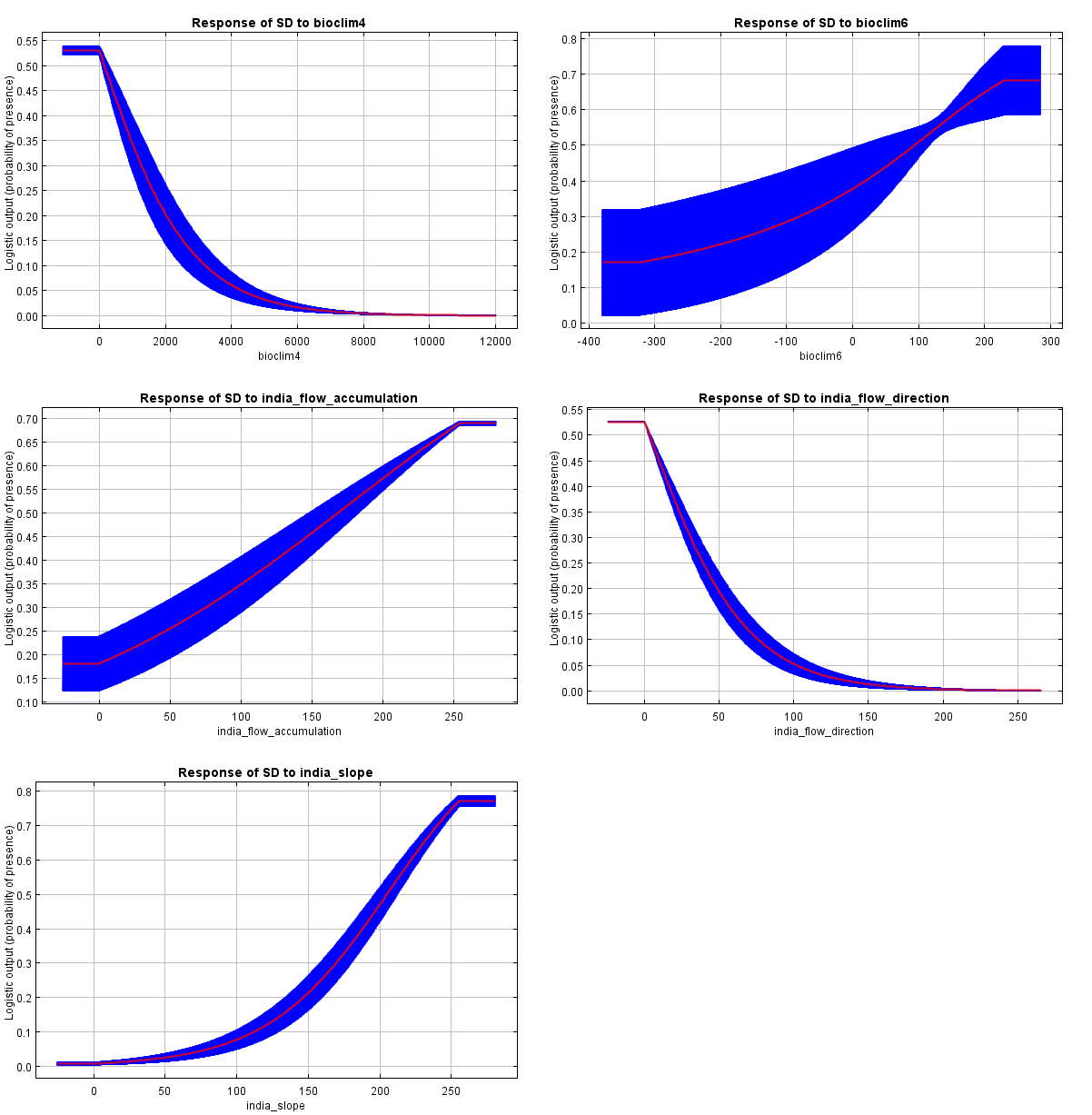


Figure S5. Response curves of top five variables of importance in *S. p. priam*.


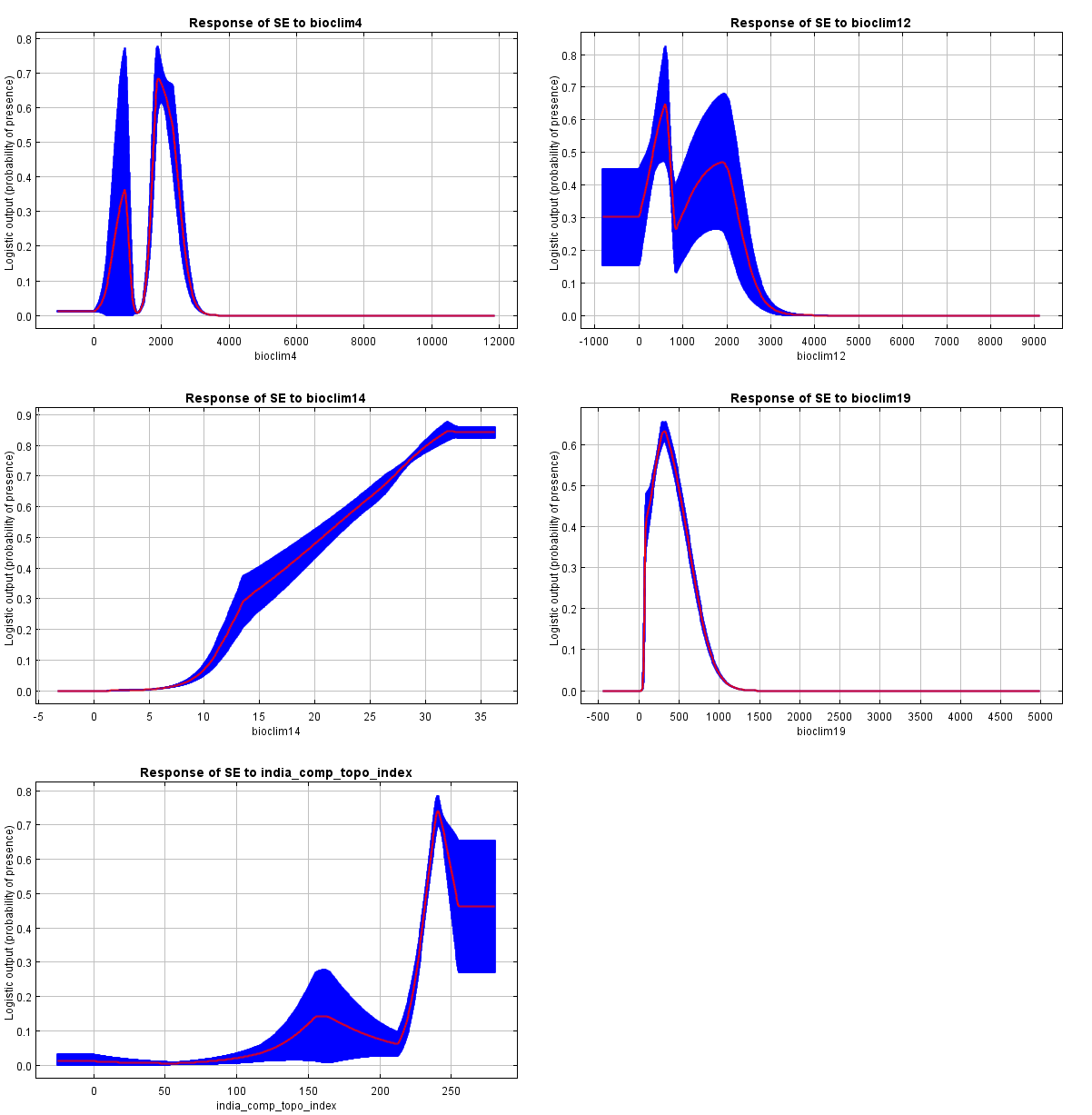


Figure S6. Response curves of top five variables of importance in *S. entellus.*


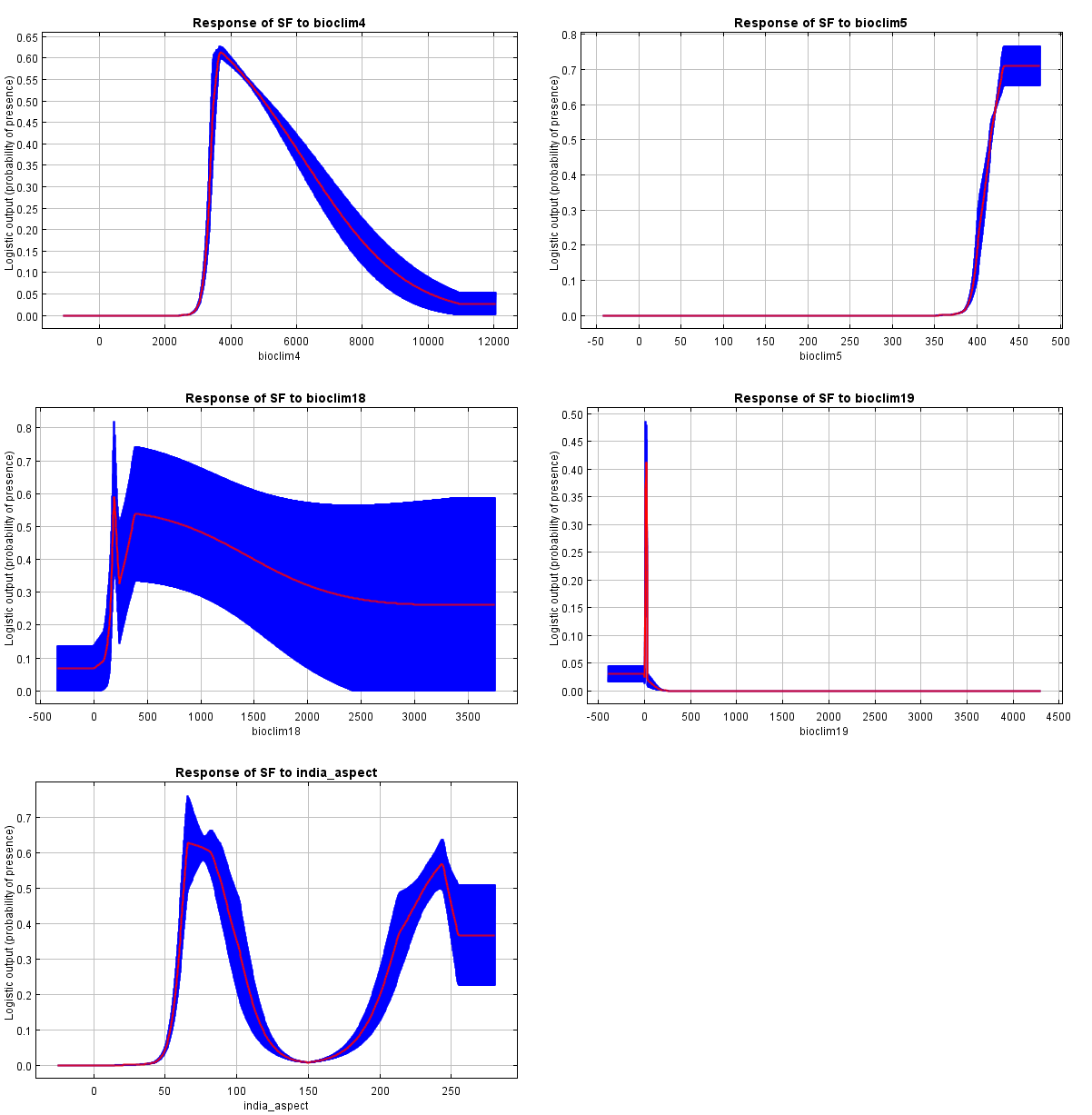


Figure S7. Response curves of top five variables of importance *S. h. iulus*.


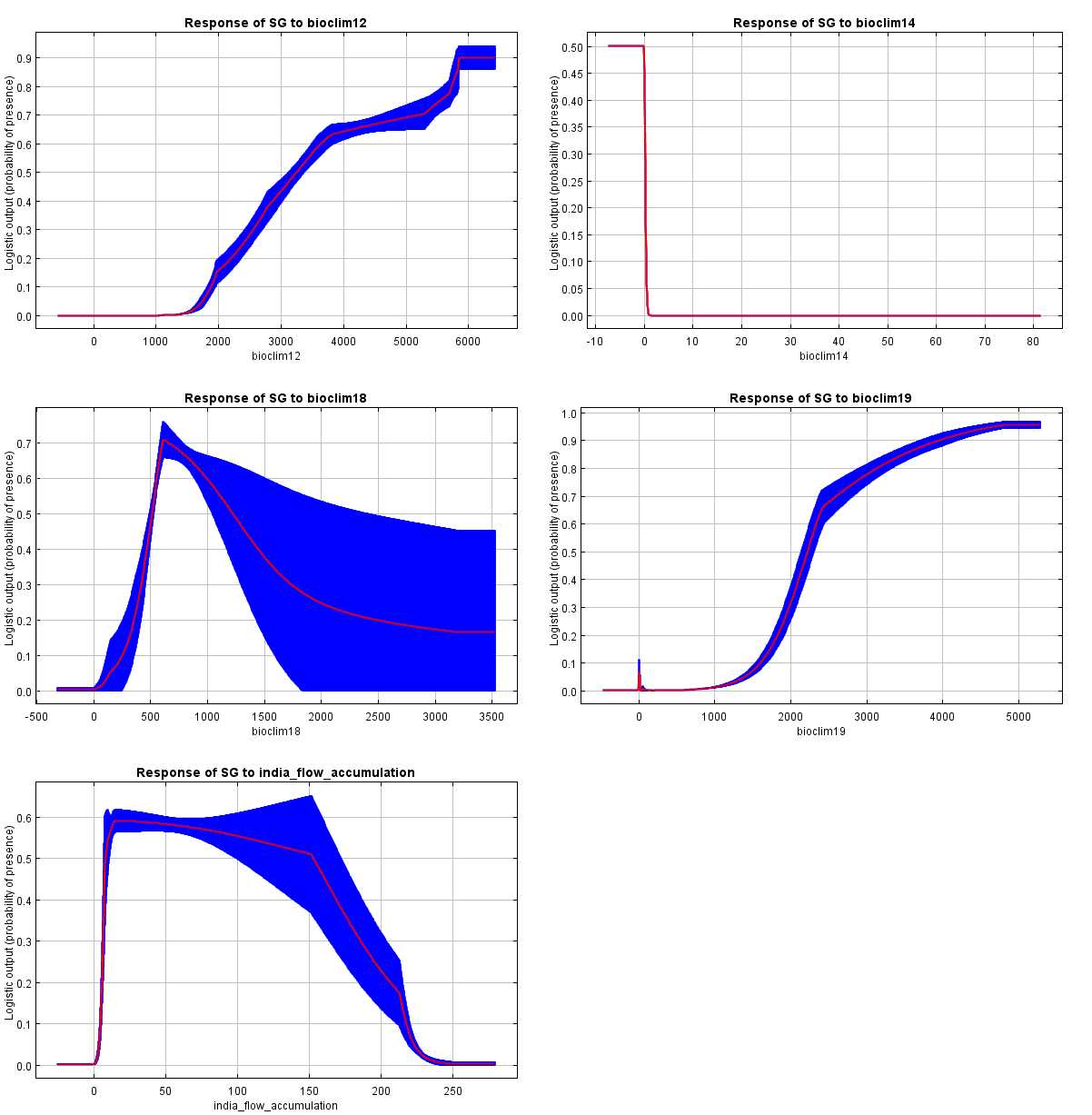


Figure S8. Response curves of top five variables of importance in *S. johnii*.


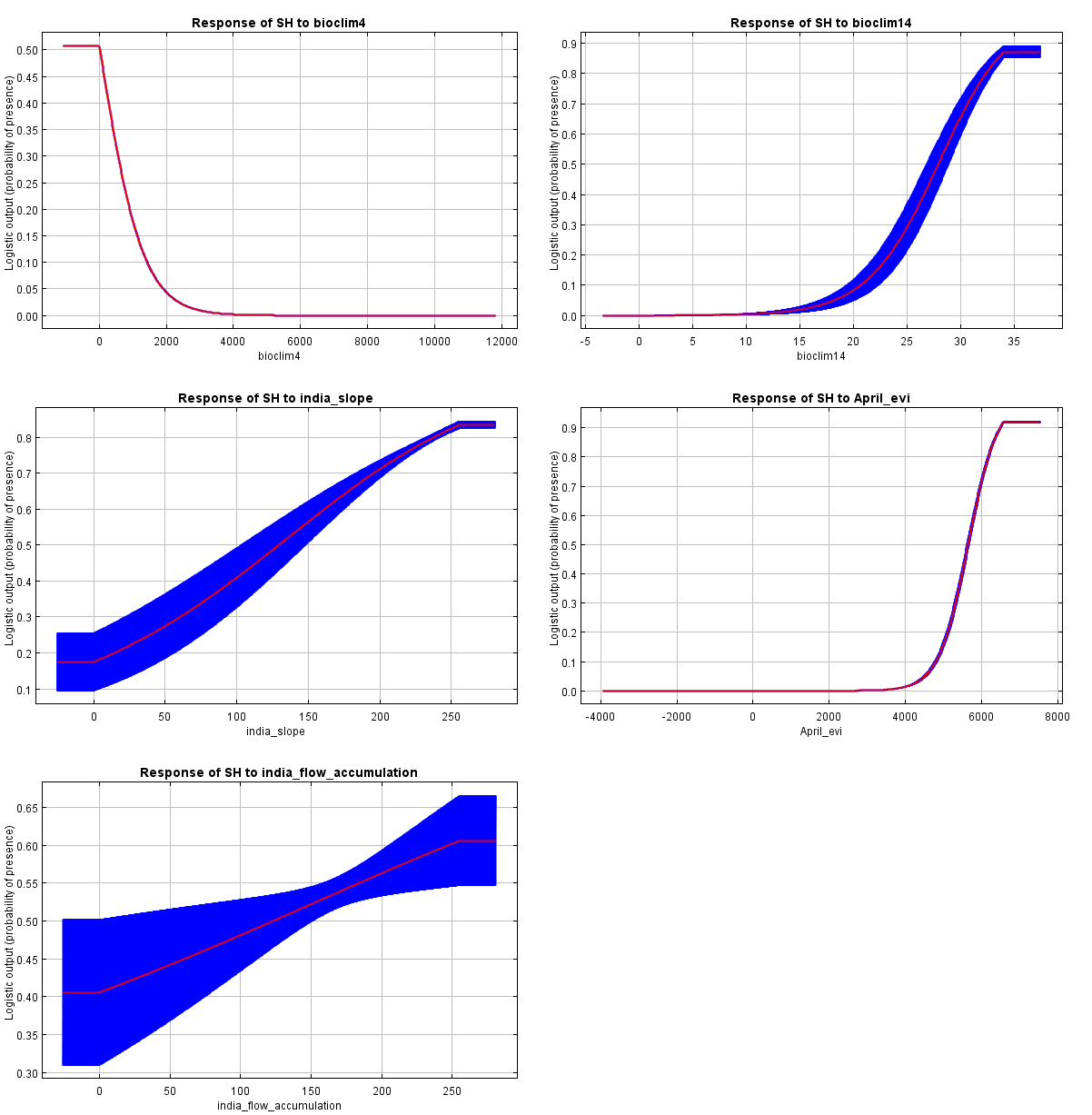

Supplement: File S1 — Combined supporting information file containing Tables S1–S4, Figures S1–S8. Table S1. Derived bioclimatic, hydrological and vegetation layers used in the present study. Each layer is with 1000 m resolution and is clipped for Indian sub-continent. Table S2. Variables with percent contribution (in bold) and permutation importance in predicted distribution of species. Table S3. Range of Environmental variables in the predicted suitability regions. Table S4. Eigenvalue and percentage variation explained in correspondence analysis. Figure S1. Response curves of top five variables of importance in S. h. achates. Figure S2. Response curves of top five variables of importance in S. h. hypoleucos. Figure S3. Response curves of top five variables of importance in S. p. anchises. Figure S4. Response curves of top five variables of importance in S. p. priamellus. Figure S5. Response curves of top five variables of importance in S. p. priam. Figure S6. Response curves of top five variables of importance in S. entellus. Figure S7. Response curves of top five variables of importance S. h. iulus. Figure S8. Response curves of top five variables of importance in S. johnii. (DOC) [file pone.0087804.s001.doc]
